# Accurate and unambiguous tag-to-gene mapping in serial analysis of gene expression

**DOI:** 10.1186/1471-2105-7-487

**Published:** 2006-11-04

**Authors:** Rodrigo Malig, Cristian Varela, Eduardo Agosin, Francisco Melo

**Affiliations:** 1 Departamento de Genética Molecular y Microbiología, Facultad de Ciencias Biológicas, Pontificia Universidad Católica de Chile, Alameda 340, Santiago, Chile; 2 The Australian Wine Research Institute, PO Box 197, Glen Osmond, Adelaide, SA 5064, Australia; 3 Departamento de Ingeniería Química y Bioprocesos, Facultad de Ingeniería, Pontificia Universidad Católica de Chile.Vicuña Mackenna 4860, Santiago, Chile

## Abstract

**Background:**

In this study, we present a robust and reliable computational method for tag-to-gene assignment in serial analysis of gene expression (SAGE). The method relies on current genome information and annotation, incorporation of several new features, and key improvements over alternative methods, all of which are important to determine gene expression levels more accurately. The method provides a complete annotation of potential virtual SAGE tags within a genome, along with an estimation of their confidence for experimental observation that ranks tags that present multiple matches in the genome.

**Results:**

We applied this method to the *Saccharomyces cerevisiae *genome, producing the most thorough and accurate annotation of potential virtual SAGE tags that is available today for this organism. The usefulness of this method is exemplified by the significant reduction of ambiguous cases in existing experimental SAGE data. In addition, we report new insights from the analysis of existing SAGE data. First, we found that experimental SAGE tags mapping onto introns, intron-exon boundaries, and non-coding RNA elements are observed in all available SAGE data. Second, a significant fraction of experimental SAGE tags was found to map onto genomic regions currently annotated as intergenic. Third, a significant number of existing experimental SAGE tags for yeast has been derived from truncated cDNAs, which are synthesized through oligo-d(T) priming to internal poly-(A) regions during reverse transcription.

**Conclusion:**

We conclude that an accurate and unambiguous tag mapping process is essential to increase the quality and the amount of information that can be extracted from SAGE experiments. This is supported by the results obtained here and also by the large impact that the erroneous interpretation of these data could have on downstream applications.

## Background

Serial Analysis of Gene Expression (SAGE) technology [1] has been described as a powerful method for genome-wide analysis of the transcriptome [2-7]. SAGE is a quantitative technique that allows the discovery of new genes and the detection of transcripts expressed at low levels. It is based on the generation of short (14 nts) nucleotide sequences denominated tags from poly(A) RNA. These tags are then concatenated serially into long DNA molecules which are sequenced in such a way that the frequency of each tag reflects the average copy number of the transcript from which it is derived [1].

A critical step in the SAGE methodology is the tag mapping process, which refers to the unambiguous assignment of an experimentally measured tag to a given transcript. Currently, the tag mapping process frequently involves the search of the observed tag sequences within the known transcriptome. Commonly employed databases available for tag mapping [8-10] use UniGene clusters [11] to map the experimental SAGE tags to the 3'-most potential tag in each expressed sequence, *i.e*. determining the UniGene cluster that most likely represents the gene from which the experimental SAGE tag was derived. Each UniGene cluster contains a collection of expressed sequences, which consists of well-characterized mRNA/cDNA sequences and expressed sequence tags (ESTs) that might represent a unique transcript. Unfortunately, this strategy allows only for the partial assignment of tags to transcripts, because the current resources for transcriptome data are incomplete for most species and organisms. Therefore, a significant fraction of the experimentally measured tags remains unidentified. In addition, there are several drawbacks of using this strategy for the mapping of SAGE tags to transcripts. First, a single gene may be represented in several clusters, resulting in ambiguous assignments. Second, EST sequences, which are the major components of the UniGene clusters, have an approximated error rate estimated at 1% (1 in 100 nts), resulting in a tag error assignment rate close to 10% [9]. Third, UniGene clusters do not contain the entire collection of transcripts and generally the genes represented in the EST databases correspond to the most abundant transcripts; therefore some tags will not be assigned (*i.e*. hypothetical and unknown genes). For example, SAGE studies in human have shown that 60% of the 14 bp tags do not have any match to sequences in the UniGene clusters [12]. The correspondence between the unmatched tags and the real transcripts was demonstrated by RT-PCR, where more than 90% of the studied unmatched tags originated from a true transcript [12]. Fourth, mapping against UniGene database does not allow the discovery of new genes, which is an important feature of SAGE data.

SAGE can be very efficient for gene discovery and annotation [3-5]. For this purpose, genome information, instead of transcriptome data, must be used in the tag-to-gene assignment process. This overcomes the problem of being limited to only those genes for which an EST has been already found. Furthermore, the genomic sequences have a low estimated error rate, of less than 0.0001% [13] and the amount of annotated genes is significantly higher than the set of expressed sequences of an organism. Therefore, genomic information is the best source for tag mapping and gene discovery by SAGE. However, the use of genomic information for tag-mapping represents a bioinformatics challenge because the complexity of large genomes makes tag uniqueness more improbable [14].

In this work, we designed a bioinformatic method that gives different confidence values to each of the multiple hits in the genome for a tag sequence. Our method allows to fully exploit the abovementioned benefits while using genomic sequences for the tag mapping process in SAGE. The confidence values were assigned according to several parameters that were obtained by the analysis of experimental SAGE tags from previous studies in yeast [15-17]. We defined this new method as hierarchical gene assignment (HGA) tag-mapping. HGA provides, in most cases, an unambiguous prediction of whether tag matches correspond to a real gene or to a region that currently is annotated as intergenic. In addition, we propose a novel and more detailed classification scheme for SAGE tags, which gives the expected confidence level of experimental tags and facilitates the processes of discovery and searching for new genes. As a proof of concept, we demonstrate the usefulness of this new method using yeast as a model organism, which results in a more complete, reliable and comprehensive assignment of experimental SAGE tags, when compared to other existing methods. We end by highlighting the benefits of using this new method on larger and more complex genomes.

## Results

### The Hierarchical Gene Assignment procedure

In this work, we describe a new method, HGA, for tag mapping in SAGE. The method combines existing knowledge of a genome sequence and its current annotation, along with known data from previous SAGE experiments, to increase the accuracy and reduce the ambiguity of the tag mapping process.

Though this methodology can be applied to any organism, we describe it here with some parameter values that have been specifically tuned for *Saccharomyces cerevisiae*. Some of these parameters are highly specific to yeast and may not be as crucial for other organisms, and viceversa.

**Figure 1:**
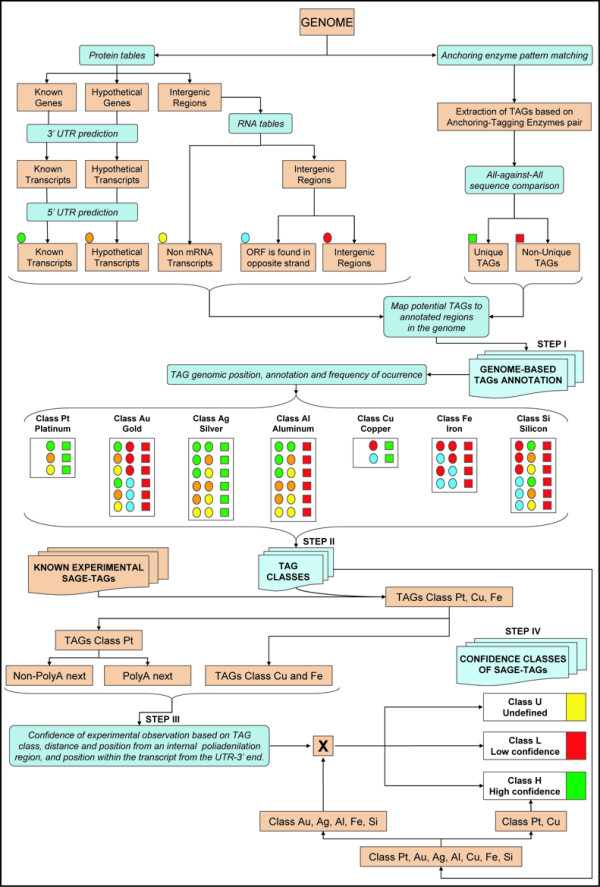
**Flowchart of the HGA method**. The method consists of four main sequential steps: ***Step 1) ***First, all virtual potential tags in the genome are extracted and compared, and the frequency of occurrence of each tag recorded, along with its particular location on the genome (top right). Then, using the most complete and updated protein and RNA tables available for the genome, in addition with the assignments and predictions of the 3' and 5' UTR regions, all potential transcripts and intergenic regions in the genome are extracted and their locations recorded. The information obtained is crossed and a detailed genome-based annotation of virtual SAGE-tags is produced. ***Step 2) ***Based on its genomic position, its annotation and its frequency of occurrence on the genome, each virtual tag is assigned to one out of seven possible classes (center). This new classification scheme helps in the assignment of tag confidence in subsequent steps. A detailed explanation of each tag class is provided in Table 1. ***Step 3) ***All known experimental SAGE-tags (Table 2) are crossed against the previously generated classification of virtual tags and only the experimental tags belonging to the classes platinum, copper and iron are selected (bottom left). The set of tags belonging to the class platinum are further subdivided into two different groups: i) those tags that map to a transcript and are not located upstream from an internal poly(A) region and ii) those tags that map to a transcript and are next to an internal poly(A) region. The genomic annotation and classification of each tag is used to determine its probability of being observed by experiment. A detailed description of the probability functions that are derived from these data is shown in Figure 2. ***Step 4) ***The tag classification generated in step 2 is crossed against the probabilities obtained in step 3 to produce a confidence assignment (high, low or undefined) for each virtual tag in the genome (bottom right). This information can be used to unambiguously map experimental SAGE-tags to annotated transcripts and/or genomic regions, along with a confidence estimation of the mapping result. A detailed explanation of the different steps used in the HGA process is provided in the main text and in methods.

HGA consists of four main steps, which are described below. A detailed flowchart of the HGA method is illustrated in Figure 1.

**Figure 2:**
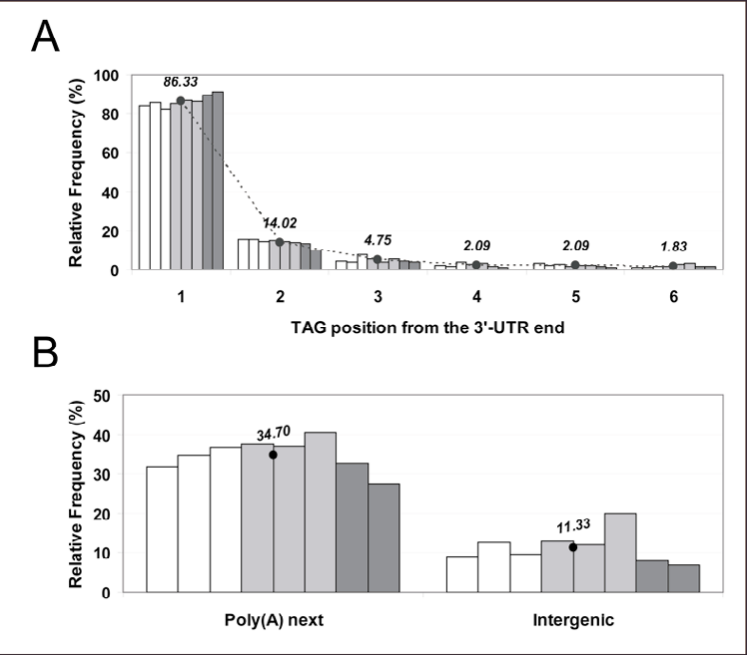
**Experimental frequency of observation of SAGE-tags in yeast**. The odds of observing a particular tag from experiment were derived from existing SAGE data for yeast upon eight different culture conditions (Table 2). From left to right in the graphs, white bars correspond to the data Var-1, Var-2 and Var-3 obtained by Varela and collaborators [17]; light gray bars correspond to the data Vel-1, Vel-2 and Vel-3 obtained by Velculescu and collaborators [15]; and dark gray bars correspond to the data Kal-1 and Kal-2 obtained by Kal and collaborators [16]. Average values from the eight data points are provided and also displayed with a black dot. (A) The odds of observing a 'non-poly(A) next' platinum tag upon its position within the transcript were derived from existing experimental SAGE data. The selected experimental tags consisted of those that were unique in the genome, mapped into known transcripts with a predicted 3'-UTR and were not categorized as 'poly(A)-next'. Platinum tags mapping into transcripts without a predicted 3'-UTR were not included to avoid errors of position estimation. (B) The odds of observing a 'poly(A) next' platinum tag was derived from existing experimental SAGE data (left panel). The selected experimental tags consisted of those that were unique in the genome, mapped into known transcripts and were categorized as 'poly(A)-next'. The odds of observing a tag mapping to intergenic positions in the genome was derived from existing SAGE data (right panel). The selected experimental tags consisted of those belonging to the classes copper and iron (Table 1).

#### Step I: Genome based extraction and annotation of potential SAGE tags

The complete genomic sequence of an organism is first searched for occurrences of the recognition site of the anchoring enzyme used in SAGE. The virtual potential SAGE-tags are then extracted by combination with a given tagging enzyme. These potential tags are then compared all-against-all in a pairwise fashion and the frequency of occurrence of each of the potential tags in the genome is determined (Figure 1, top right). The transcript tables containing the known genomic annotations are used by the HGA method to map the occurrence of genes in different locations of the studied genome (Figure 1, top left). In the case of *Saccharomyces cerevisiae*, its protein tables only specify the coding regions of each gene (verified, dubious and hypothetical transcripts) and do not contain the assignment of the untranslated regions (UTRs) at the 5' and 3' ends. Therefore it was necessary to assign them. The precise assignment of these regions is particularly relevant in the case of the 3'-UTRs, because it is expected that a significant fraction of experimental SAGE-tags will be obtained from these regions. With better knowledge of the transcriptome, a larger fraction of the UTRs can be accurately assigned. For most model organisms, a large number of expressed sequence tags (ESTs) are available even though only a small fraction of full length cDNAs is known. Therefore, the precise assignment of UTRs for most of the coding genes is not possible. For yeast, about half of the known genes have a predicted 3'-UTR with high confidence. These are mainly due to the identification of downstream polyadenylation signals [18]. For those cases where the 3'-UTR is not available, a fixed length is assigned (see Methods).

Once all 3'-UTRs are assigned for each coding gene, the HGA method proceeds to complete the annotation of the coding transcripts with the assignment of the 5'-UTRs. In the case of yeast, little is known about the 5'-UTRs, but we used the length of 100 nts because more than 95% of the experimental tags that map into the 5'-UTRs are observed at an upstream distance from the initial codon of the open reading frame (ORF) of less than 100 nts [19]. At this point, known and hypothetical coding transcripts are annotated as accurately and completely as possible (Figure 1, top left).

After the assignment of complete coding transcripts to the genome, the RNA tables are used to map and assign the non-mRNA transcripts (see methods). This feature of the HGA method is new, because previous works in SAGE have not explicitly used the non-coding transcripts to map experimental tags. Though most non-coding transcripts do not contain poly(A) tails, and thus should not be observed in SAGE experiments, a recent study has shown that some ribosomal RNAs are polyadenylated in yeast, even in the absence of a canonical polyadenylation signal [20]. Furthermore, priming to internal poly(A) regions of RNA molecules during reverse transcription occurs at a high frequency [21]. Thus, we included non-coding RNAs in the annotation of the yeast genome to be used for mapping of virtual SAGE-tags.

Once all transcripts have been assigned, the remaining intergenic regions of the genome are categorized into two types, depending on whether an annotated transcript is present in the complementary strand or not (Figure 1, top middle). When the procedure described above completes, the genome information is organized into five categories,: 1) Known transcripts, 2) Hypothetical transcripts, 3) Non-mRNA transcripts, 4) Intergenic regions where a transcript is found on the opposite strand, and 5) Intergenic regions on both strands.

#### Step II: Definition of tag classes and features

The structured genome information generated above is crossed against all the potential tags, generating a genome based annotation of virtual SAGE-tags. The resulting virtual tags are categorized into one of seven classes, depending on the genomic position, annotation and frequency of occurrence of each virtual SAGE-tag in the genome (Figure 1, center). A detailed definition for each tag class is given in Table 1. This new proposed tag categorization facilitates the inference of not only the assignment confidence, but also the potential knowledge that can be extracted from a particular tag (*ie*. its potential contribution for the tasks of gene discovery, genome annotation and generation of knowledge about indirect regulation of gene expression by antisense RNAs).

**Table 1:** Class definition of virtual genomic SAGE tags

**TAG CLASS**		
**ID**	**NAME**	**DESCRIPTION**

**Pt**	**Platinum**	TAG is unique in the genome and it matches a transcript
**Au**	**Gold**	TAG is not unique in the genome and it matches a transcript, but other occurrences of this TAG always match intergenic regions
**Ag**	**Silver**	TAG is unique in the genome but it matches two or more overlapping transcripts located at the same genomic region
**Al**	**Aluminum**	TAG is not unique in the genome, it matches a transcript, but other occurrences of this TAG also match another transcript located at a different genomic region
**Cu**	**Copper**	TAG is unique in the genome and it matches an intergenic region
**Fe**	**Iron**	TAG is not unique in the genome, it matches an intergenic region, but other occurrences of this TAG always match an intergenic region
**Si**	**Silicon**	TAG is not unique in the genome, it matches an intergenic region, but other occurrences of this TAG match a transcript

As an important complement for this new tag classification scheme, the HGA method also incorporates two additional tag features, which are intended to reduce some potential distortions that can affect the interpretation of SAGE results. First, all continuous stretches of eight or more adenines within each annotated transcript are recorded to account for oligo-dT priming to internal poly(A) regions of RNA molecules during reverse transcription. It has been demonstrated that this process occurs at a high frequency, causing that about 12% of ESTs are truncated due to internal poly(A) priming [21]. Therefore, those tags mapping within a transcript and situated near and upstream of an internal polyadenylation site are labelled as 'poly(A) next'. Otherwise, they are labelled as 'non-poly(A) next'. Second, the effect of splicing and its potential impact on tag sequence generation is considered. Tags mapping onto a transcript at an intron-exon boundary are labelled 'potential-splice-tags'. In this case, a virtual splicing is generated in the computer and the new tag sequence that would match the spliced and mature transcript is produced and recorded as 'spliced-tag'. Each 'spliced-tag' inherits the classification previously assigned to its former 'potential-spliced-tag'. In those cases where a new recognition site for the anchoring enzyme used in SAGE is generated after splicing, the new virtual tag sequences are generated and recorded. These tags are labelled as 'potential-new-tags' and their corresponding class is calculated *de novo*. The remaining tags are labelled as 'non-spliced-tags'.

#### Step III: Extraction of probability values for tag observation from experimental data

The resulting tag classification, along with the abovementioned additional tag features, are used to select particular tags from the genome, the occurrences of which are searched for among known experimental SAGE-tags obtained previously and described in the literature for the studied organism. Table 2 shows the known SAGE data currently available for yeast. Selected tags belong to three different classes: platinum (Pt), copper (Cu) and iron (Fe) (see Table 1 for details), and they are chosen because these tags can be unambiguously assigned to a unique transcript or intergenic region in the genome. Thus, the probability that a given potential tag with some specific characteristics would be observed by experiment can be obtained. For those tags mapping onto a single transcript, with a unique sequence in the genome (Pt class tag) and labelled as 'non-poly(A) next', the likelihood to observe them experimentally, as a function of their mapping position from the 3'-UTR can be calculated. In this case, we obtained several probability values depending on the transcript position the tag maps, thus incorporating the effect of partial digestions with the anchoring enzyme in SAGE experiments. The obtained probability function for yeast is shown in Figure 2A. On the other hand, for Pt class tags labelled 'poly(A) next', a single probability value is derived from experimental data. This value gives the likelihood to obtain an experimental tag as consequence of oligo-d(T) priming to an internal poly(A) region during the cDNA synthesis (Figure 2B). Finally, copper (Cu) and iron (Fe) tag classes correspond to tags that map to an intergenic region of the genome; the former is unique and the latter represents multiple matches in the genome. In both cases, the frequency of occurrence of these tag classes in the experimental data indicates the probability that a tag arose from an intergenic region, according to the current genome annotation of a specific organism (Figure 2B).

**Table 2:** Experimental SAGE-TAG libraries from Yeast

**ID**	**UNIQUE TAGs**	**DESCRIPTION**
**Var-1**	908	Mid-exponential phase during the fermentation process [17]
**Var-2**	725	Early stationary phase during the fermentation process [17]
**Var-3**	641	Late stationary phase during the fermentation process [17]
**Vel-1**	2,226	Logarithmic growth [15]
**Vel-2**	2,341	S phase-arrested [15]
**Vel-3**	2,154	G2/M boundary arrested [15]
**Kal-1**	1,268	Wild type oleate-grown cells [16]
**Kal-2**	649	Pip2/oaf1 mutant oleate-grown cells [16]

To summarize, all the potential tags are assigned with a value according to the genomic regions they map and to their specific features. For example, the tags mapping into a certain transcript will have different values according to the transcript position and the proximity of internal poly(A) sequences, whereas the tags mapping to intergenic regions have a single value.

#### Step IV: Odds ratios for confidence assignment of virtual SAGE-tags

**Figure 3:**
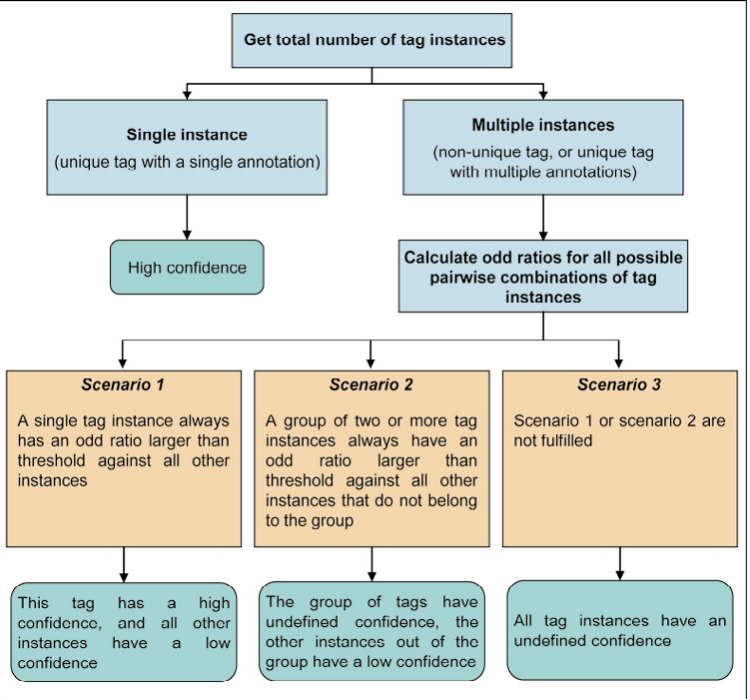
**Flowchart of confidence assignments by the HGA method**. For the case of unique tags in the genome that have a single annotation, a high confidence is assigned. For those tags that either are non-unique in the genome or do have two or more annotations, a more complex algorithm is used to assign a confidence class to them. First, all tag instances in the genome are selected. Then, the assignment of tag confidences is carried out as explained in the figure.

**Figure 4:**
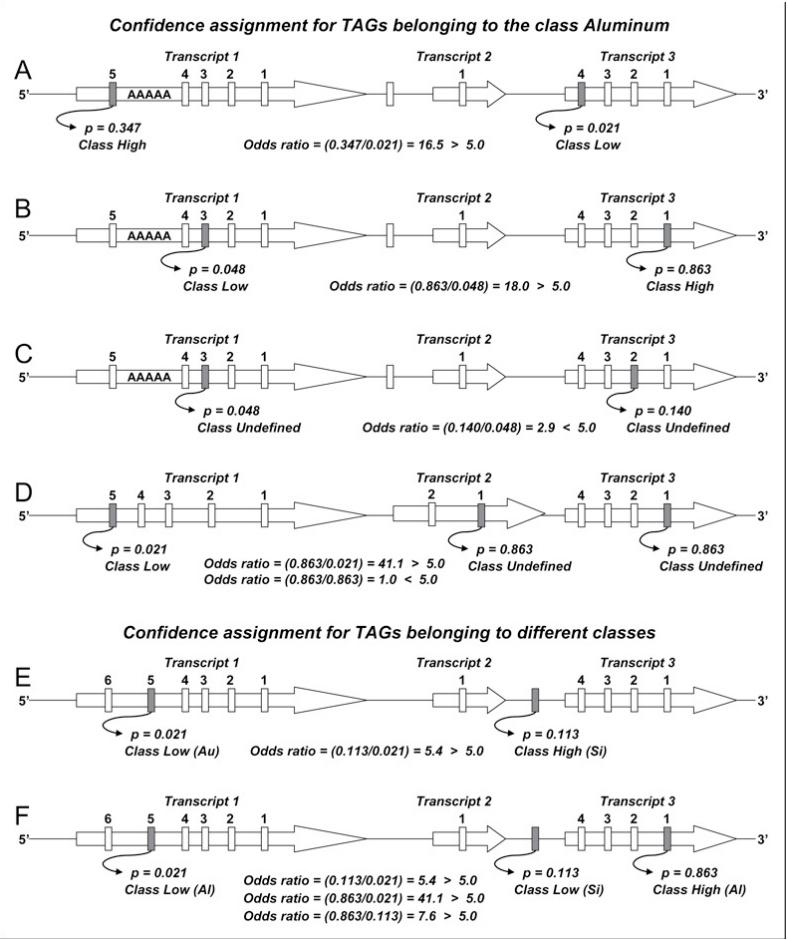
**Examples of tag confidence assignments by the HGA procedure**. Different scenarios of confidence class assignments for virtual genomic tags are shown. In panels A through D, confidence class assignments for aluminum tags are exemplified. In panel E and F, confidence class assignments for tags belonging to different classes are shown. Unique tag sequences always have a high confidence and are only included to provide a genomic context (empty rectangles). Genomic tags sharing the same sequence are illustrated by a dark gray colour. In the case of tags mapping into an annotated transcript, the position within the transcript is also shown. Poly(A) next tags are identified by the presence of a downstream poly(A) region. Before the assignment is performed, the probability of experimental observation for each relevant virtual tag instance is obtained from Table 3, depending on the observed features of each tag. Then, all possible odds ratios using the largest value in the numerator are calculated. In those cases where a particular tag instance exhibits an odd ratio larger than 5.0 when compared against all other genomic instances of the tag sequence, a high confidence class is assigned to that tag. The remaining tag instances are assigned either a low or an undefined confidence class. The undefined confidence class is assigned when the odd ratio between two instances is lower than the threshold of 5.0. A detailed flowchart of the tag confidence assignment process is shown in Figure 3.

The estimated probability functions described above are then crossed against all virtual genomic tags, to obtain a tag confidence assignment for each potential virtual SAGE-tag in the genome (Figure 1, bottom right). The tag confidence gives the odds that an experimental tag is properly assigned to a virtual genomic tag and is represented in the HGA method by one of three possible classes: 1) High confidence, 2) Low confidence, or 3) Undefined confidence. The class 'High confidence' means that the tag has a high probability of being correctly assigned. All virtual tags that are unique in the genome and have a single annotation are assigned to this class. In those cases where a tag sequence occurs two or more times in the genome, the odds ratios among all instances of the tag are calculated (Figure 3). The odds ratios for all possible pairwise combinations of tags belonging to the different categories generated in this work for the yeast organism are shown in Table 3. A high confidence has been arbitrarily defined by us as being at least five times more probable than all possible alternative assignments. This odds ratio figure equivalent to five is a parameter of the method and therefore could be easily modified. The class 'Low confidence' is the opposite of the class 'High confidence', meaning that there is an alternative assignment for the same tag sequence that is at least five times more probable. The class 'Undefined confidence' is assigned to those cases where there is not a single instance of a tag occurrence in the genome that can be assigned to the class 'High confidence' (*ie*. among all occurrences of a tag sequence that is observed multiple times in the genome, there is not a case where a particular virtual tag always has an odds ratio equal or higher than five when compared against all other instances). In these cases, the tags could still be ranked based on the odds ratios that they exhibit, which is provided by the annotation generated by the HGA method. Some examples illustrating how the tag confidence assignment process is carried out by the HGA method are shown in Figure 4.

**Table 3:** Odds ratios for hierarchical genome assignments of SAGE tags

		**TAG**							
		
		**Transcript without internal polyadenylation sites**						**Poly(A) next**	**Intergenic**
		
		**TAG position from 3'-UTR end**							
		
		**1**	**2**	**3**	**4**	**5**	**>= 6**	**Any**	**N.A.**
	
	Probability	0.863	0.140	0.048	0.021	0.021	0.018	0.347	0.113
**1**	0.863	1.00	**6.16**	**18.17**	**41.31**	**41.31**	**47.16**	2.49	**7.64**
**2**	0.140	0.16	1.00	2.95	**6.71**	**6.71**	**7.66**	0.40	1.24
**3**	0.048	0.06	0.34	1.00	2.27	2.27	2.60	0.14	0.42
**4**	0.021	0.02	0.15	0.44	1.00	1.00	1.14	0.06	0.18
**5**	0.021	0.02	0.15	0.44	1.00	1.00	1.14	0.06	0.18
**>= 6**	0.018	0.02	0.13	0.39	0.88	0.88	1.00	0.05	0.16
**Poly(A) next**	0.347	0.40	2.48	**7.30**	**16.61**	**16.61**	**18.96**	1.00	3.07
**Intergenic**	0.113	0.13	0.81	2.38	**5.41**	**5.41**	**6.17**	0.33	1.00

Odds ratios between all possible pairs of tag types are calculated based on the eight probabilities of experimental observation shown in Figure 2. These probabilities are included in the third row and in the second column of the table, while the tag type descriptions are provided in the first two rows and in the first column of the table. Odds ratios are calculated by considering in the numerator the probability of the tag type shown in each row; the corresponding probability of the tag type shown in each column is used in the denominator. Odds ratios higher than 5.0 are highlighted with bold type font.

### Annotation of virtual genomic SAGE-tags from yeast by the HGA method

We applied the HGA method to the full genome of *Saccharomyces cerevisiae *(Table 4). We found that 80% of the 76,826 potential virtual tags were unique in the genome (platinum and copper tag classes). 54% of the potential virtual tags mapped onto intergenic regions and the remaining 46% to transcripts. About 60% of these intergenic tags had an annotated transcript on the opposite strand, though this was expected, given the high coding density present in the yeast genome.

**Table 4:** Annotation of virtual SAGE-tags for yeast by the HGA method

	**All TAGs**		**Unique TAGs**	
		
	**Frequency**	**Percentage**	**Frequency**	**Percentage**
***Classes***				
Platinum (Pt)	28,948	37.68	28,948	43.40
Gold (Au)	2,075	2.70	815	1.22
Silver (Ag)	363	0.47	143	0.21
Aluminum (Al)	4,139	5.39	1,580	2.37
Copper (Cu)	31,844	41.45	31,844	47.74
Iron (Fe)	6,417	8.35	2,037	3.05
Silicon (Si)	3,040	3.96	1,336	2.00
***Confidences***				
High	62,780	81.72	62,780	94.12
Undefined	11,082	14.42	3,923	5.88
Low	2,964	3.86	0	0.00
***Transcript context***				
UTR-5'	1,669	4.70	1,481	4.70
ORF (CDS)	28,645	80.63	25,049	79.56
UTR-3'	5,211	14.67	4,956	15.74
***Intergenic and transcripts***				
Intergenic	41,301	53.76	35,217	52.80
Transcript opposite	30,208	39.32	27,407	41.09
Transcript full	35,172	45.78	31,143	46.69
Partial transcript	353	0.46	343	0.51
Total transcripts	35,525	46.24	31,486	47.20
***Introns***				
Intron	380	1.07	212	0.67
Non-intron	35,145	98.93	31,274	99.33
***Poly(A) next***				
Poly(A) next	1,606	4.52	1,541	4.89
Non-poly(A) next	33,919	95.48	29,945	95.11
***Non coding RNAs***				
Non-mRNAs	367	1.03	203	0.64
***Splicing***				
Spliced	13	0.02	12	0.02
***Position within the transcript***				
Position 1	6,415	18.06	6,156	17.48
Position 2	5,733	16.14	5,423	15.40
Position 3	4,943	13.91	4,306	12.23
Position 4	4,054	11.41	3,542	10.06
Position 5	3,217	9.06	2,787	7.91
Position 6	2,537	7.14	2,186	6.21
Position 7	1,965	5.53	1,672	4.75
Position 8	1,516	4.27	1,267	3.60
Position 9	1,163	3.27	972	2.76
Position 10	894	2.52	754	2.14
***Total***				
Total	76,826	100.00	66,703	100.00

When the HGA method was applied to the yeast genome, 82% of the potential virtual tags were classified with high confidence, thus reducing the ambiguity to 2% of the potential tags, out of the 20% tags that are not unique in the genome. In other words, HGA increased unambiguous annotations by 10%.

Most of the virtual tags that mapped onto annotated transcripts are located within the coding region (81%), or on the 3'-UTRs (15%) and a small fraction of the tags were found at the 5'-UTRs (5%). As should be expected, these figures correlate with the observed lengths of these elements.

The total number of virtual tags shows an inverse linear relationship to its position within the transcript, as expected, based on the fact that position number correlates with distance from the 3'-UTR end, which is directly related to the probability of finding a downstream recognition site for the anchoring enzyme used in SAGE.

Only a small fraction of the virtual tags map onto annotated introns (1%) and non-coding RNAs (1%). Very few tags map onto exon-intron boundaries (0.02%), accounting for a total of 13 new tag sequences generated by splicing.

When we considered potential unique virtual tag sequences within the genome, most of the results described above remain unchanged (Table 4, right columns). The only tag features that showed major differences with the results given above involved the tag classes and confidences. In the first case, the fraction of tags belonging to the platinum and copper classes, which represent unique tags in the genome, increased. The other tag classes decreased at least two fold in proportion to the full genome annotation, because most of the non-unique tags are repeated two times in this genome. In the case of tag confidence classes, the total number of high confidence tags remained the same, but there was a 12% increase in the fraction of this confidence class because the total number of virtual tags is lower when most of non-unique tags are discarded. The proportion of undefined confidence tags decreased about 9%, because several instances of repeated tags were eliminated. Low confidence tags were not assigned because their high confidence counterparts were assigned and the low confidence tags discarded. In summary, when only the potential tag sequences that could be observed by a SAGE experiment are considered, a potential gain of about 3% of the high confidence class is achieved by the HGA method. In the case of the yeast genome, this means that, when using this method, about 2,000 additional experimental SAGE-tags could be assigned with a high confidence to a single gene or genomic region. The complete annotation of virtual SAGE-tags from the yeast genome generated when using this method is available as supplemental material (see methods).

### Mapping of experimental SAGE-tags from yeast against the HGA-based annotation

We collected all published data available from SAGE experiments in yeast (Table 2). We then used the annotation of virtual SAGE-tags generated by the HGA method for this organism to map these experimental SAGE-tags (Table 5). Several of these results contributed to validate the HGA method. First, about 82–90% of the experimental tags were mapped onto transcripts and not onto intergenic regions; as expected for an organism with a complete genome annotation. Most of these tags belonged to the classes platinum, aluminum and gold. A large fraction of these tags mapped to coding and 3'-UTR regions of transcripts, and a few mapped to 5'-UTR regions. For all cases, more than 91% of the experimental tags mapped belong to the high confidence class according to the HGA-based annotation. All these facts suggest that the HGA method is reliable.

**Table 5:** Mapping of experimental yeast SAGE-tags against the HGA-based annotation

	**Varela [17]**		**Velculescu [15]**		**Kal [16]**	
			
	**Frequency**	**Percentage**	**Frequency**	**Percentage**	**Frequency**	**Percentage**
***Classes***						
Platinum (Pt)	884	69.44	2,187	72.88	993	75.17
Gold (Au)	54	4.24	134	4.47	68	5.15
Silver (Ag)	5	0.39	12	0.40	5	0.38
Aluminum (Al)	112	8.80	255	8.50	139	10.52
Copper (Cu)	183	14.38	304	10.13	82	6.21
Iron (Fe)	26	2.04	75	2.50	23	1.74
Silicon (Si)	9	0.71	34	1.13	11	0.83
***Confidences***						
High	1,177	92.46	2,756	91.84	1,204	91.14
Undefined	96	7.54	245	8.16	117	8.86
***Transcript context***						
UTR-5'	15	1.52	29	1.19	14	1.26
ORF (CDS)	574	58.16	1,699	69.80	693	62.15
UTR-3'	398	40.32	706	29.01	408	36.59
***Intergenic and transcripts***						
Intergenic	218	17.12	413	13.76	116	8.78
Transcript opposite	140	11.00	250	8.33	60	4.54
Transcript full	1,041	81.78	2,561	85.34	1,188	89.93
Partial transcript	14	1.10	27	0.90	17	1.29
Total transcripts	1,055	82.88	2,588	86.24	1,205	91.22
***Introns***						
Intron	6	0.61	5	0.21	1	0.09
***Poly(A) next***						
Poly(A) next	70	7.09	189	7.76	73	6.55
***Non coding RNAs***						
Non-mRNAs	12	1.22	7	0.29	4	0.30
***Splicing***						
Spliced	1	0.08	1	0.03	4	0.30
***Total***						
Total	1,273	100.00	3,001	100.00	1,321	100.00

There are several interesting new features that are presented in this work concerning experimental SAGE data. First, though there are few instances, tags mapping onto introns are observed in all the SAGE experiments examined. Second, a significant fraction of tags located near an internal poly-(A) region within a transcript are observed in all SAGE experiments reported. Third, in all experiments, SAGE-tags mapping onto non-coding RNAs are observed. Almost all these cases consist of tags belonging to the class 'non-poly(A)-next' and mapping to the first position within the transcripts, suggesting that typical polyadenylation occurs at the 3' end of these transcripts (data not shown, available as supplemental material). Fourth, analogous to what was observed for introns, spliced-tags are observed in all SAGE experiments. This is the first time that experimental SAGE-tags are mapped onto virtual and potential spliced-tags from a genome. Fifth, a significant fraction of experimental SAGE-tags map onto regions in the genome that currently are annotated as intergenic. Though this has already been observed, it must be mentioned that it is for the first time that this analysis is carried out by considering the confidence of the assigned tags, and thus the figures obtained here should be more accurate. These intergenic tags could represent new genes not yet described in yeast. Using the HGA-based annotation they can now be easily ranked according to their estimated confidence, which will facilitate and optimize the experimental planning of the gene discovery process. Finally, a large fraction of the experimental tags that map onto an intergenic region has an annotated transcript on the opposite strand. These tags could correspond either to new genes or to new regulatory elements such as antisense RNA [22,23]. The detailed genome mapping of known experimental yeast SAGE-tags, generated by searching against the HGA-based annotation, is available as supplemental material.

### Comparison of the previous experimental SAGE-tags assignments with the results obtained by the HGA-based annotation

**Figure 5:**
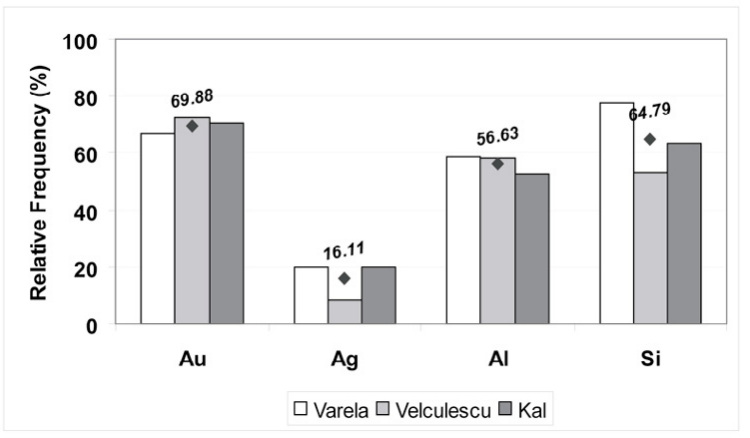
**Gain of unambiguous assignments by the HGA method**. The percentage of cases where other authors assigned two or more genes to a tag and the HGA method assigned the same experimental tag to a single gene are plotted independently for each tag class and for each publication of SAGE experiments in yeast. White bars correspond to the cumulated data from Var-1, Var-2 and Var-3 experiments [17]; light gray bars correspond to the cumulated data from Vel-1, Vel-2 and Vel-3 experiments [15]; and dark gray bars correspond to the cumulated data from Kal-1 and Kal-2 experiments [16]. In the case of the tags belonging to the class silicon, the HGA method assigned them as mapping to a single intergenic region whereas other authors mapped these tags to those occurrences within annotated transcripts.

We compared the gene assignments of experimental SAGE-tags carried out by the authors of the different SAGE experiments reported in yeast (Table 2) with those generated here by the HGA method for the same experimental data (Table 6). About 8–10% of the ambiguous assignments by other authors (including unique tags) were unambiguously classified by the HGA method. In these cases, the authors of the SAGE experiments assigned a tag to two or more genes and the HGA method assigned the same tag with a high confidence to a single gene. When the gain of unambiguous tag assignments by the HGA method is calculated considering only those cases with ambiguity (*i.e*. those with several assignments by other authors), independently for each tag class, the obtained figures are highly significant (Figure 5). For the gold, aluminum and silicon tag classes, 57 to 70% gain of unambiguous assignments was achieved. The gain of about 16% obtained for the tags of class silver is low because in most of these cases all tag instances mapped to the first positions of each transcript. However, this tag class is less abundant and thus it only has a small impact on the absolute gain.

**Table 6:** Comparison of experimental SAGE-tags assignments

	**Varela [17]**		**Velculescu [15]**		**Kal [16]**	
			
	**Frequency**	**Percentage**	**Frequency**	**Percentage**	**Frequency**	**Percentage**
***Assignments performed by the authors***						
Single assignment	1,067	83.82	2,491	83.01	1,074	81.30
Multiple assignments	206	16.18	510	16.99	247	18.70
***Assignments performed by the HGA-based annotation***						
Single assignment	1,177	92.46	2,756	91.84	1,204	91.14
Multiple assignments	96	7.54	245	8.16	117	8.86
***Gain of unambiguous assignments by the HGA-based annotation***						
Gain	110	8.64	265	8.83	130	9.84
***Conflicting assignments (between other authors and the HGA-based annotation)***						
Class High	69	5.42	154	5.13	115	8.71
Class Platinum	41	3.22	90	3.00	36	2.73
Class Copper	28	2.20	64	2.13	79	5.98
***Total number of assignments***						
Total	1,273	100.00	3,001	100.00	1,321	100.00

We also found some conflicting assignments of unique tags to single genes between former SAGE experiments and those performed here (Table 6). In the cases of tags belonging to the platinum class, the authors assigned a different gene than the HGA method. The low number of conflicting assignments for these tags validates the HGA method. For tags belonging to the copper class, the literature assigned a single gene and the HGA method mapped the tag onto an intergenic region. In both cases, most of the conflicting assignments are due to the different length of 3'-UTRs used by the authors (a fixed length of 500 nts) and by the HGA method (a variable length, with a maximum value of 370 nts for those cases with unknown 3'-UTRs). This issue and its significance are discussed in the next section.

## Discussion

In this work, we present a novel bioinformatics method called hierarchical gene assignment or HGA, for the accurate tag-to-gene mapping process in SAGE. The HGA method has two major advantages compared to other previously described approaches [8-10]: 1) a new tag classification scheme, useful for the initial identification of SAGE-tag features and to infer the capabilities of tags for gene assignment and gene discovery. 2) a confidence assignment for potential SAGE-tags in the genome. These two advantages allow to minimize the number of unambiguous assignments of experimental tags to genomic regions in SAGE.

### Improvements in tag mapping by the HGA method

Several new features that improve the accuracy and completeness of the tag mapping process in SAGE have been incorporated by the HGA method, and are detailed below.

First, instead of using only the coding regions of known and hypothetical genes, we assigned, as precisely as possible, the 3' and 5' UTRs, thus generating more accurate putative transcripts. Mature and immature transcripts were generated, by considering exon-intron boundaries, thus keeping and using all the relevant available genomic information and annotation. When no information about UTRs was available for a given gene, we used a fixed maximal length estimated from experimental data. It is noteworthy that most conflicting assignments of unique tags observed between HGA and other authors' assignments resulted from the large length of 3'-UTRs previously used by these authors [15-17] (Table 6). The precise assignment of 3'-UTRs is critical for accurate mapping of SAGE tags. This is one of the key contributions of the HGA method to data analysis in SAGE.

Second, non coding RNAs, in addition to known and hypothetical genes, were also included in the genomic annotation. Though the amount of tags mapping to these transcripts is low (Table 5) and most of them do not contain a poly(A) tail at the 3'-end, it is important to include them to maximize the analyzing power of SAGE.

Third, tags mapping to intergenic regions in the genome, where an annotated transcript is found in the opposite strand, were also considered in the HGA method. These supposedly intergenic tags, if experimentally observed, could account for unknown elements, such as antisense RNA. We showed that a significant fraction of these tags were observed in SAGE experiments with yeast (Table 5), even though *S. cerevisiae *constitutes one of the best annotated genomes available today. If EST data were used to map experimental SAGE-tags, this information would not be obtained. Hence, a method that considers these elements explicitly in the annotation process would accelerate the discovery of new regulatory elements. The identification of regulatory elements of this kind is important for a complete and accurate interpretation of the gene expression patterns.

Fourth, by using genomic information in the tag mapping process, the HGA method identifies tags mapping onto regions where no gene annotations exist in either of the DNA strands. In this work, we demonstrated that a significant fraction of these tags were observed in SAGE experiments with yeasts (Table 5). It is again worth mentioning that, although gene annotation for yeast is quite complete when compared to other organisms and that a significant fraction of the genome is currently annotated as coding, a large amount of intergenic tags experimentally observed by SAGE suggests that many unknown transcripts are still to be discovered. This observation agrees with previous results obtained from DNA microarray experiments [24].

Fifth, the combined use of genomic information along with the generation of new putative splicing tags not explicitly available in the genome sequence, allows a more accurate estimation of tag uniqueness and, therefore, of potentially ambiguous mappings.

Sixth, the inclusion of internal poly(A) regions within annotated transcripts as possible reverse transcription initiation sources is another important feature of the HGA method. This was included because recent EST data analyses have shown that a significant fraction of the reverse transcription processes are initiated at internal poly(A) regions of more than 8 consecutive adenines [21]. The results with poly(A)-next tags from SAGE experiments described here (Table 5) confirmed this feature, strongly supporting its consideration in accurately mapping experimental tags onto genes. Interestingly, about 5% of the virtual tags mapped onto annotated transcripts are classified as 'Poly(A)-next', accounting for a total of 1,606 occurrences in the genome, suggesting that these instances should not be overlooked when mapping experimental tags onto a genome. Moreover, in the case of Pt class and 'poly(A) next' tags, the position of the tag within the transcript should not be relevant for the probability that a tag was experimentally observed. We verified this and found that, as expected, a small and insignificant effect was observed. Further, given the low proportion of 'poly(A) next' tags occurrence, the inclusion of a position dependent probability for 'poly(A) next' tags would translate into a small number of experimental observations for several positions within the transcript, which would add noise to the actual estimated probability functions. When sufficient experimental data from SAGE experiments is available, a position dependent probability function for Pt class and 'Poly(A) next' tags should be derived.

Seventh, the new definition of tag classes considered by the HGA method (Table 1) facilitates the understanding of the tag origin from a genome along with an initial estimation of the confidence that this tag could be observed in a SAGE experiment.

Finally, the calculation of tag probabilities from experimental data based on the new tag classification, along with other tag features, allows the HGA method to get the odds or confidence that a tag would be experimentally observed when several instances of a tag sequence are present in the genome. This constitutes the core of the HGA method and one of the most important contributions of this work to reduce the number of unambiguous tag assignments in SAGE. In addition, we also demonstrated that about 20% of the experimental tags mapping onto a transcript are located from the second tag position and above. If this information is not considered in the tag-to-gene mapping process, a substantial fraction of the experimental tags will be missed. Finally, it is important to note that even in those cases where the ambiguity could not be completely removed, the HGA method could reduce the number of possible assignments, thus reducing the overall ambiguity for a particular tag with multiple occurrences in the genome.

### Parameters depending on genome annotation

The score of intergenic tags is strongly dependent on the quality of the genome annotation. In poorly annotated genomes, intergenic tags will have a higher probability of being observed by the HGA methodology. This is a desirable feature for tag probability estimation in the discovery of new genes. In yeast, 11.3% of all experimental SAGE-tags obtained to date and searched against the current annotation of the yeast genome map into an intergenic region, suggesting that new coding or non-coding transcripts are still to be discovered. This figure will be even larger for poorly annotated genomes.

### Significance of HGA-based annotation for tag mapping on complex genomes

In this work, we achieved an 8–10% increase in unambiguous tag assignments when considering all experimental yeast SAGE-tags (Table 6). This improvement rose as high as 70% when only those tags with multiple matches to the genome were considered (Figure 5). In yeast, with a relatively small genome with a size of about 12 million base pairs, the fraction of unique tags of 14 nts accounts for 87% of the potential virtual genomic tags. Therefore, 8–10% increase in unambiguous assignments has limited interest. However, when larger genomes with sizes of billions base pairs are considered, the fraction of unique tags of 14 nts was significantly reduced to 10% (see below our preliminary results for *Xenopus *genome). Therefore, the main problem of using genome sequences for tag-to-gene assignment in long genomes is that with their increased size and complexity tag uniqueness and unambiguous tag-mapping becomes increasingly difficult. It is in these cases that HGA would be most useful, because it will significantly reduce unambiguous tag mappings.

Long-SAGE has been proposed to reduce the ambiguity of tag mapping for large genomes [14,25]. However, there are some major drawbacks of using this new technique, such as the higher costs involved, lower efficiency of tag sequencing and a significantly increased sequencing error rates for tags of 20–21 nts, estimated to occur in 20% of the experimental tags derived from long-SAGE experiments [26]. Whether SAGE or Long-SAGE technology should be used for genome-wide analysis is still a matter of debate. The generation of SAGE tags with 30 bases implies a 3-fold increase in sequencing cost when compared with 10 bp tags, a high increase in cost for an 8% increase in unambiguous mapping when UniGene databases are used [27]. Contrary to this, others have demonstrated that an increase in the length of the tag is crucial for tag uniqueness when the genome is used for mapping [14].

To better assess or estimate which SAGE methodology should be used, we performed some bioinformatics analysis of the virtual tags from the recently released *Xenopus tropicalis *genome (data not shown, work in progress). This genome has 1.5 billion base pairs, half the size of the human genome and 125 times larger than the yeast genome. Our preliminary analysis of all potential tags for *X. tropicalis *genome showed that tag uniqueness for short (14 nts) virtual tags is around 9.1%, small compared to the 80.6 % of uniqueness for the long (21 nts) virtual tags. When a histogram of occurrence for each tag sequence was constructed, we found that 60% of the virtual short tags have less than 7 matches to the genome and 90% of the tags have less than 20 matches. This low number of genome hits per tag for a significant amount of the potential short tags suggests that the use of HGA tag-mapping should allow proper tag-to-gene assignment when typical SAGE technology with tags of 14 nts is used. More importantly, when we built a virtual reference database for SAGE-tags from *X. tropicalis *genome using only some of the parameters involved in the HGA method described here, we have found that 40% of the short virtual tags are included in the high confidence class. This indicates that after using the HGA method, a significant fraction of the short tags will be unambiguously mapped with a high confidence to the genome and represents a total potential gain of unambiguous assignments of about 31%. This figure is significantly higher than the 8–10% obtained for yeast, though its estimation for *X. tropicalis *was based on the virtual genomic tags instead of the experimental SAGE-tags, where this figure will increase, as it was shown for yeast (Table 4). In addition to this, though in several cases the ambiguity will not be completely eliminated, it will be significantly reduced. All this suggests that the HGA method will be most useful for tag-mapping in large and complex genomes, providing a highly-efficient and low-cost alternative to Long-SAGE.

However, more complex and larger genomes can pose new challenges that were not faced in this work with yeast. Among these challenges are the large number of alternative splicing transcripts that are more frequently observed in complex genomes. This constitutes a problem for the accurate estimation of position-dependent tag probability functions. Therefore, some modifications to the HGA methodology proposed here will be required depending on the particular genome.

We are currently adapting the HGA method to annotate as accurately as possible all virtual SAGE-tags for the genomes of xenopus, arabidopsis, mouse and human, and these results will be published elsewhere.

## Conclusion

In this work, we have presented a novel bioinformatics method for tag mapping in SAGE. The method has been tested and validated using experimental SAGE data from *Saccharomyces cerevisiae *organism. Based on the results obtained, we draw the following conclusions:

1) A significant increase of unambiguous assignments for experimental SAGE-tags in yeast is achieved when using this method (Figure 5).

2) Using a genome-based annotation of virtual SAGE-tags like the one described here shows that a significant fraction of experimental SAGE-tags comes from intergenic regions, from partially digested cDNA, from the opposite strand of annotated transcripts, and from truncated cDNAs (Figure 2, Table 5).

3) In all SAGE experiments reported for yeast, tags map onto introns, exon-intron boundaries and onto non-coding RNAs (Table 5).

4) In all SAGE experiments reported for yeast, it was observed that the largest fraction of tags map to the coding regions of transcripts and not to the 3' UTR elements (Table 5).

## Methods

### Source for genome sequence information

The full genome sequence of the *Saccharomyces cerevisiae *organism was obtained from the July 2005 release available at the Saccharomyces genome database (SGD) web site [28]. The full genome sequence included the 16 nuclear chromosomes and the single mitochondrial chromosome. The original source of the complete genomic sequence used in this work is available as supplemental material at our web site [29].

### Source for genomic annotation

The July 26^th ^2005 release of the genomic annotation available at the SGD web site was used [28]. This original source file was filtered by selecting only those records that contained in the feature type field one of the following keywords: ORF, intron, rRNA, tRNA, snoRNA, snRNA and ncRNA. Other field identifiers such as origin of replication or telomere region were discarded. The original source of the complete genomic annotation table used in this work is available as supplemental material at our web site [29].

### Building a virtual genomic restriction map for the extraction of a virtual library of genomic SAGE-tags

Several computer programs in C++ and ANSI C languages were written to perform specific tasks. First, the full DNA sequence of each chromosome in the genome was fragmented in a computer into all possible overlapping oligonucleotide sequences of a length of 14 base pairs (bp) using the computer program *subsequence*. This process was carried out for the forward and reverse DNA strands, and the results concatenated into a single file. Second, the 14 bp sequences were filtered, selecting only those that matched the pattern CATG at their 5' end. This procedure resulted in a total of 76,516 tags that represent the theoretical product of a complete genome digestion by using a combination of the NlaIII and BsmFI restriction enzymes (ie. all potentially observable SAGE tag sequences). In this process, the position and strand where each tag was found in the genome were stored. For this purpose, we set up a new computer program called *pattern*. Finally, an all-against-all pairwise tag comparison without mismatches was performed and the frequency of occurrence for each tag sequence at the different genomic positions on both strands stored with another computer software: *freqtag*. All these computer programs for the LINUX operating system are freely available as supplemental material at our web site [29].

### Putative coding RNAs from the genomic annotation of ORFs and from predictions of 5' and 3' UTRs

Only the records containing the 'ORF' word in the feature type field identifier from the filtered genome annotation table were considered for the annotation and prediction of 5' and 3' UTRs. This restriction yielded a total of 6,591 ORF candidates for the 3' and 5' UTR assignments. The 3'-UTR ends were first assigned to all those cases that cross-matched the previously described annotation [18]. The latter contained a total of 3,141 3'-UTRs, out of which 132 were not assigned. Most of these unassigned 3'-UTRs corresponded either to pseudogenes and transposable elements or their systematic names did not match any ORF. A total of 2,937 3'-UTRs were defined based on that UTR annotation. The 3'-UTRs for the remaining 3,654 ORFs were assigned by the following procedure: 1) A fixed length of 370 nts was assigned if the first position of the next annotated transcript on the same chromosome and strand in the 3' direction was located more than 370 nts away from the end position of the ORF. This length was chosen because more than 95% of the 3'-UTRs described in yeast are equal or shorter than this length [18]. A total of 2,752 3'-UTRs were assigned with a fixed length of 370 nts. 2) If the total number of nucleotides available between the end of the ORF and the initial position of the next transcript was equal or shorter than 370 nts, then the 3'-UTR end of the ORF was assigned at the previous nucleotide of the initial position of the next transcript. 824 3'-UTRs were assigned in this way. 3) For ORFs where their 3' ends were contained within another annotated transcript element, no 3'-UTRs were assigned (*ie*. the 3'-UTR was defined with a length of zero nts; its end position was therefore assigned to the same position of the end of the annotated ORF). A total of 78 3'-UTRs with a length of zero nts were assigned.

After all 3'-UTR assignments were completed, the 5'-UTRs were assigned as follows: as 5' UTRs of most of yeast ORFs are unknown, a fixed 5'-UTR length of 100 nts was assigned for all those ORFs where the previous annotated transcript was located at a larger distance than 100 nts. In those cases where an ORF was located upstream, then the previously assigned 3'-UTR end position was considered as the end position of the upstream ORF. 100 nts was chosen because more than 95% of the experimental tags that map into the 5'-UTR are observed at an upstream distance from the initial codon of the ORF of less than 100 nts [19]. A total of 5,112 5'-UTRs of 100 nts were assigned. Then, if the total number of nucleotides available between the end of the upstream annotated transcript and the initial position of the ORF was equal or shorter than 100 nts, then the initial position of the 5'-UTR was assigned to the next nucleotide of the end position in the previous transcript. A total of 1,214 5'-UTRs were assigned with a length shorter than 100 nts, but larger than zero. In the case of the ORFs where their 5' ends were contained within another annotated transcript element, no 5'-UTRs were assigned (*ie*. the 5'-UTR was defined with a length of zero nts and therefore its initial position was assigned to the first nucleotide corresponding to the first codon of the annotated ORF). 265 5'-UTRs with a length of zero nts were assigned.

### Mapping virtual genomic SAGE-tags to putative coding RNAs and to non-coding RNAs

In addition to the putative coding transcripts, all non-coding RNAs available in the genomic annotation table were selected. Then, all the virtual genomic SAGE tags were mapped against these annotated elements, based on their genomic positions. Both complete and partial tag matches to a transcript were recorded. A complete match was defined when the virtual tag was totally contained within the transcript. A partial tag match was defined only if the previous condition was not fulfilled and if the most 5' nucleotide was contained within the transcript; otherwise the virtual tag was defined as intergenic. The same criterion described above was used to define those tags mapping to introns. A total of 775 introns are currently annotated within known transcripts in the yeast genome. The virtual tags partially mapping an exon-intron boundary were annotated as such, but in these cases the potential new tags not present in the genomic sequence that could be obtained by the splicing process were also generated and stored into the database. Only 13 potential new tags fulfilling these conditions were generated.

### Mapping intergenic virtual genomic SAGE-tags to the opposite strand of putative coding RNAs and to non-coding RNAs

All virtual tags defined as intergenic in the previous step were assessed for their occurrence in the opposite strand of an annotated transcript. All virtual tags located within an intergenic region but fully contained in the opposite strand of an annotated transcript were also annotated as such. These tags could be important for the discovery of new interference RNA elements [22].

### Mapping virtual genomic SAGE-tags within putative coding RNAs and to non-coding RNAs that are located near downstream internal poly-A regions

Virtual tags located within a transcript, but not at the most 3' end position, were observed with a high frequency experimentally due to internal poly(A) priming of oligo-d(T) primer during reverse transcription [21]. Therefore, for all virtual tags mapping within a transcript, the current position from the 3' end was recorded. Then, all poly(A) regions of 8 or more consecutive adenines that were found within any annotated transcript were recorded. Finally, all those tags mapping within a transcript at the second position or above from the 3' end, where a downstream poly(A) region was located at up to 800 nucleotides and another virtual tag was not found in between, were defined as virtual tags next to an internal poly(A) site.

### Assignment of classes to virtual SAGE-tags

The class of virtual tags was defined based on three characteristics: 1) the frequency of occurrence of the tag sequence in the genome (whether it is unique or not), 2) the number of annotations for the tag, and 3) the type of annotations of the tag. Using these three distinct features we have defined seven different quality classes for any virtual tag occurring in the genome. The naming and definition of tag classes are described in Table 1.

### Definition of confidence for virtual SAGE-tags based on their unambiguous assignment to transcripts and intergenic regions in the genome

The confidence of virtual tags was defined based on a combination of the tag class, the frequency of occurrence in the genome and the position of mapping within the transcript. All potential tags in the genome were classified as high, low, or undefined confidence. High confidence tags correspond to those that can be unambiguously assigned to a single known gene or to a single intergenic position in the genome. Low confidence tags correspond to those that should not be visible by experiment, because another tag of identical sequence but mapping at a different location in the genome should be. Undefined confidence tags correspond to those that are fuzzy and cannot be assigned clearly to a single gene or intergenic region in the genome. The procedure that we used to define tag confidence is as follows:

First, the probability of observation by experiment was calculated for those tags belonging to the class platinum, copper and iron. In the case of the class platinum, the tags were subdivided into two groups depending if the tag was or was not located upstream from an internal poly(A) region. In the case of those tags that were not defined as next to an internal poly(A) site we calculated their frequency of occurrence at different positions in the transcript from the 3'-UTR end, using the currently known experimental SAGE data (Figure 2A). To minimize possible errors in the probability estimations versus position in the transcript, we selected only those experimental tags mapping to transcripts with a known 3'-UTR annotation. The total number of occurrences of tags upon different positions within a transcript was recorded and added up. At the same time, the total number of potential platinum tag sites in the same transcripts at different positions was recorded. Then, the fraction of these platinum tags was calculated for each position within a transcript (Figure 2A). The calculations were performed independently for each of the eight known experimental SAGE libraries from yeast (Table 2) and the average was calculated. In the case of those tags platinum defined as next to an internal poly(A) site, a similar procedure was carried out. However, due to the lack of enough experimental observations, a position independent probability of occurrence was calculated (Figure 2B, left panel). Finally, the probability of experimentally observing tags mapping to intergenic regions in the genome was calculated by counting the total number of tags belonging to the classes copper and iron, and then dividing this figure by the total number of experimental tags (Figure 2B, right panel). These calculations were also performed independently from each experiment and then the average probability was calculated. The different obtained probabilities of experimental observation considered in this work, along with a pairwise calculation of all possible odds ratios is shown in Table 3.

Second, all tags belonging to the classes platinum and copper were defined as high confidence. In the case of non-unique tags in the genome, or in the case of unique tags with multiple annotations (*ie*. tags belonging to the silver class), an assessment of the confidence was carried out based on some tag features and on the previously obtained probabilities. The probability of occurrence is obtained from the previous data for all the instances of a particular tag sequence. If an instance of a tag maps to an intergenic region in the genome, then the probability of observing copper and iron tag sequences is used. If an instance of a tag maps into a transcript, then it is first evaluated if it is located upstream from an internal poly(A) region or not. In the case of the former, the probability of a tag mapping upstream from an internal poly(A) region is used; otherwise, the probability is obtained based on the position within the transcript that the tag is found. Once the individual probabilities are obtained for all the instances of the tag sequence, a pairwise comparison table is built, which contains all-against-all odds ratios among the instances. Then, if a single instance of a tag has odd ratios higher than 5.0 against all other instances, this tag is defined as a high confidence tag, and all other instances as low confidence. Otherwise, all tag instances are assigned an undefined confidence.

### Building a complete library of known experimental SAGE-tags from yeast

We compiled all experimental information available from SAGE experiments in yeast. These include three independent works [15-17], accounting for a total of 8 different experimental data points (Table 2).

### Mapping experimental SAGE-tags to the virtual library of SAGE-tags

The mapping process of experimental SAGE-tags against the virtual library was performed by assigning to the experimental tag the annotation of the high confidence virtual tag, when possible; otherwise, the experimental tag was assigned to multiple transcripts and/or intergenic regions with an undefined confidence.

## Availability and requirements

A web server that uses the HGA-based annotation described in this manuscript for the genomic mapping of experimental SAGE tags from yeast or for the exploration of the virtual SAGE-tags on this organism has been implemented and it is freely accessible.

**Project home page: **

**Operating systems: **MacOSX, Linux, Windows

**Programming language: **C++, ANSI C, PERL, PHP, MySQL

**Other requirements: **none

**License: **none

**Any restrictions to use by non-academics: **none
